# COVID-19’s impact on bioanalytical labs

**DOI:** 10.4155/bio-2021-0103

**Published:** 2021-08-10

**Authors:** Amy Lavelle, Janine Micheli

**Affiliations:** 1 Immunochemistry R&D, PPD, 2244 Dabney Road, Richmond, VA 23230, USA

**Keywords:** bioanalysis, clinical trials, COVID-19, NAAT, SARS-CoV-2 antigen, serology

## Abstract

One year later, the impact of the COVID-19 pandemic on business practices, supply chains, timelines and analytical needs for COVID-19 clinical trials have been felt across the bioanalytical community, as therapeutics may now require SARS-CoV-2 antigen and serological testing.

The COVID-19 pandemic has presented unique challenges to the bioanalytical community. One year later, the impact of the pandemic on business practices, supply chains, timelines and project prioritization have been felt across the industry. Due to travel and personnel restrictions, laboratories have adapted their day to day lab operations and clinical trial practices. Logistically, supply chains have been affected, resulting in reduced availability of lab consumables, reagents, personal protective equipment (PPE) and biological matrices. The analytical needs for COVID-19 clinical trials are more varied than traditional therapeutic candidates as well, as each therapeutic can require pharmacokinetic, immunogenicity, biomarker and SARS-CoV-2 antigen and serological testing.

## How it started

In the spring of 2020, the first devastating global pandemic in recent history began to unfold. Along with the rest of the world, pharmaceutical companies closed their laboratories and industry work was paused as we adjusted to the changes COVID necessitated to attempt to mitigate the pandemic. Changes in daily operations included new approaches to employee presence, scope of work, outsourcing and audits.

Arguably, on-site personnel restrictions had greatest impact on pharmaceutical companies. Laboratories require on-site bench scientists and clinical staff for projects to progress through the drug-development pipeline. Most large Pharma companies were completely shut down temporarily. Upon reopening, many companies operated using a multiple shift model for daily operations and shifted a significant portion of the workforce to remote work. Safety restrictions required laboratory areas to implement social distancing, increase disinfecting procedures, require self-monitoring for COVID-19 symptoms and increased PPE requirements. COVID-19 contact tracing and quarantine protocols meant that work was frequently paused for weeks at a time from possible COVID exposure and COVID infections. Limited staff also meant that work previously performed in-house at Pharma companies, such as smaller studies or early discovery/preclinical work was now outsourced to CROs or even halted to allow resources to shift to larger or higher priority trials. Clinical sites also experienced reduced enrollment and limited staff. These challenges combined to reduce expected trial sizes and created significant delays in study timelines.

Laboratories and clinical sites were also affected by the impact lockdowns had on supply chains for laboratory consumables and reagents. Nearly all supplies, from biological matrices, reagents, equipment and even PPE, saw shortages and increased delivery lead times due to increased demand, manufacturing issues as well as staff to process orders. In response, the pharmaceutical and contract pharmaceutical industries worked to secure critical supply inventory by adopting strategic 6-month supply stockpiles and establishing relationships with new or secondary vendors. International border restrictions and custody flow also resulted in delays in inventory shipments.

Travel restrictions resulted in a shift from onsite to virtual audits. This included both sponsor and regulatory audits. This also meant adjusting standard audit practices to a virtual format. While the scope of audits remained relatively unchanged, there were changes in practice to allow data sharing over secured networks rather focus on hard copy data in face-to-face audits. While these audit adjustments did not notably impact milestones or timelines, it was a change in how the industry was accustomed to working.

## How it is going

A year of adjustments due to COVID-19 has resulted in new ‘standard’ business practices for the pharmaceutical industry including socially distanced and limited laboratory staff, increased remote job roles, new audit practices and changes to supply chain management. COVID-19 vaccinations are becoming more widespread, which could eventually mean a return to fully staffed laboratories and clinics as well as the resumption of in-person audits. However, most companies are considering permanently relocating some onsite staff to fully remote roles. This could lead to overhead cost reductions and increased space for laboratory personnel. Virtual audits also seem to be a preferred change because of COVID practices, particularly for low-risk or initial vendor audits.

Another more permanent concern and more significant impact to business practice are global supply chain issues. There were severe disruptions in inventory availability as early in the pandemic. This has led companies to become more cautious and pro-active in ensuring they have adequate stock for long-term needs as there continues to be limited availability for some items across multiple vendors.

Clinical study timelines seem to be minimally impacted by the pandemic. As clinical sites have reopened and patients feel comfortable coming into clinics and hospitals, studies that were delayed or paused have resumed operations. Sample testing impacts include enrollment issues, inclusion of COVID-19 screening in protocol designs and study setup logistics such as remote sampling. Many trials adopted a decentralized model, incorporating remote sampling and more patient-centric sampling efforts. These changes have reduced the delays witnessed early in the pandemic that were due to clinical site shutdowns and quarantines. The COVID-19 pandemic has demonstrated the need and patient desire for a shift to the decentralized clinical trial model.

## The response

The response to the COVID-10 pandemic by the pharmaceutical and healthcare industries has resulted in the development of over 1000 vaccine and therapeutic candidates, and almost 5000 clinical trials being conducted currently [1]. This explosion in innovation, along with a shift to greater outsourcing by pharmaceutical and biotechnology companies, has led to an exponential demand for CRO bioanalytical capabilities from *de novo* analytical method development to high throughout, rapid turnaround testing.

In addition to the logistical challenges that bioanalytical laboratories have faced during this pandemic, there has been an unprecedented need to develop a suite of bioanalytical methods to support COVID-19 diagnosis as well as determine the efficacy and safety of novel therapeutics and vaccines. The breadth of bioanalytical methods that have been required is staggering: pharmacokinetic, immunogenicity and biomarker assays are needed for each new therapeutic. Nucleic acid amplification test (NAAT) and antigen testing are needed for COVID-19 diagnosis and clinical trial enrollment. Finally, SARS-CoV-2 serology testing is required for post-COVID-19 exposure identification and pharmacodynamic characterization of therapeutic and vaccine candidates [2].

Generally, NAAT and antigen testing is being performed in clinical laboratories on automated platforms or as point-of-care testing. Many large clinical testing laboratories have developed these tests in-house or have adopted assay platforms from large, reputable suppliers such as Roche’s Cobas platform. At the time of writing, there were 241 molecular and 23 antigen tests that have received an emergency use authorization (EUA) from the US FDA [3]. The USA alone has administered almost 500 million COVID-19 tests since the start of the pandemic [1].

Serology testing for anti-SARS-CoV-2 antibodies has been more problematic than NAAT or antigen testing for several reasons: serology tests may have significant specificity issues due to cross reactivity with previous exposure to other coronaviruses, serology testing must characterize, which subclasses of immunoglobulins are being detected, for example, IgG, IgM, IgA or total Ig and serology assays need sufficient sensitivity to produce meaningful results, particularly when they are used as a pharmacodynamic or exploratory end point for therapeutic products. As an illustration to the difficulty in developing a reliable serology test compared with NAAT and antigen testing, there are only 76 serology assays with EUAs and two serology assays have had their EUAs revoked due to specificity and sensitivity issues [4]. Serology assays are developed on various platforms including lateral flow assays for point-of-care or home testing, as well as immunoassays that use numerous platforms and technologies that can be performed in high and medium complexity laboratories.

The FDA guidance on drug development for drugs and biologics on COVID-19 prevention and treatment specifically requires that COVID-19 therapeutic trials assess anti-SARS-CoV-2 antibodies and a testing strategy for identifying COVID-19 cases [5]. Additionally, subgroup analyses stratified by immune response may prove valuable in elucidating the efficacy of vaccine and therapeutic candidates. The resource requirements to support the numerous clinical trials has led the bioanalytical industry to search for analytical methods that have high throughput and that are sufficiently sensitive and specific to provide meaningful data within a reasonable timeframe.

Our Immunochemistry laboratory developed multiple Ig subclass (IgG, IgM and IgA) serology assays and a neutralizing antibody assay to support therapeutic candidate studies. It was clear during method development of these assays that nonspecific binding was a significant challenge that needed to be overcome for SARS-CoV-2 serology assays. Common approaches for eliminating background or nonspecific interactions were evaluated such as blocking, reagent and washing step optimizations; however, no assay condition was found that could eliminate nonspecific binding. There were two options for eliminating the nonspecific interactions, dilute the samples until no nonspecific binding was observed or correct for the nonspecific binding. Sample dilution was quickly rejected as a solution because the required dilution would result in an assay sensitivity in the µg/ml range, which was not sufficient sensitivity to support therapeutic studies.

Our solution consisted of a unique method of sample handling that eliminated nonspecific binding and resulted in nanogram/ml sensitivity and greater than 90% clinical specificity and sensitivity. (manuscript preparation in progress).

To further increase our sample throughput, we are currently adapting our serology assays to the 384-well format, which will increase our throughput sixfold. The strategic use of higher throughput formats plus automated and semi-automated solutions has allowed our lab to keep pace with the increased method development and sample analysis demand due to the pandemic.

## Future perspective

The COVID-19 pandemic has been a challenge for the bioanalytical scientist, as we have never been more resource constrained, while at the same time needing to rapidly increase testing capability and capacity. The lessons learned over the last year have taught us the importance of adaptability, strategic inventory management and the importance of innovation in a time of crisis. While the pandemic has had a devastating effect on the world, the results of all the efforts of the pharmaceutical, CRO and healthcare industries could lead to meaningful and impactful changes to the medical field. These changes could lead to novel therapeutics being developed and made available to the public for numerous diseases.
